# Proteomic aptamer analysis reveals serum biomarkers associated with disease mechanisms and phenotypes of systemic sclerosis

**DOI:** 10.3389/fimmu.2023.1246777

**Published:** 2023-09-11

**Authors:** Francesca Motta, Antonio Tonutti, Natasa Isailovic, Angela Ceribelli, Giovanni Costanzo, Stefano Rodolfi, Carlo Selmi, Maria De Santis

**Affiliations:** ^1^ Department of Biomedical Sciences, Humanitas University, Pieve Emanuele, Italy; ^2^ Rheumatology and Clinical Immunology, Istituti di Ricovero e Cura a Carattere Scientifico (IRCCS) Humanitas Research Hospital, Rozzano, Italy

**Keywords:** connective tissue disease, interstitial lung disease, proteomics, aptamer technology, immunology

## Abstract

**Background:**

Systemic sclerosis (SSc) is an autoimmune connective tissue disease that affects multiple organs, leading to elevated morbidity and mortality with limited treatment options. The early detection of organ involvement is challenging as there is currently no serum marker available to predict the progression of SSc. The aptamer technology proteomic analysis holds the potential to correlate SSc manifestations with serum proteins up to femtomolar concentrations.

**Methods:**

This is a two-tier study of serum samples from women with SSc (including patients with interstitial lung disease - ILD - at high-resolution CT scan) and age-matched healthy controls (HC) that were first analyzed with aptamer-based proteomic analysis for over 1300 proteins. Proposed associated proteins were validated by ELISA first in an independent cohort of patients with SSc and HC, and selected proteins subject to further validation in two additional cohorts.

**Results:**

The preliminary aptamer-based proteomic analysis identified 33 proteins with significantly different concentrations in SSc compared to HC sera and 9 associated with SSc-ILD, including proteins involved in extracellular matrix formation and cell-cell adhesion, angiogenesis, leukocyte recruitment, activation, and signaling. Further validations in independent cohorts ultimately confirmed the association of specific proteins with early SSc onset, specific organ involvement, and serum autoantibodies.

**Conclusions:**

Our multi-tier proteomic analysis identified serum proteins discriminating patients with SSc and HC or associated with different SSc subsets, disease duration, and manifestations, including ILD, skin involvement, esophageal disease, and autoantibodies.

## Introduction

Systemic sclerosis (SSc) is a connective tissue disease characterized by a pathogenetic triad composed of microangiopathy, immune system activation, and fibrosis. SSc is associated with significant morbidity and mortality largely dependent on the early detection of organ involvement including skin, lung, gastrointestinal tract, and heart (1–3).

Serum autoantibodies are the only available biomarkers for precision medicine in SSc as anti-centromere antibodies (ACA) are commonly observed in the limited form (lcSSc) and associated with an increased risk of pulmonary arterial hypertension (PAH) while anti-RNA polymerase III antibodies are linked to scleroderma renal crisis and anti-topoisomerase I (anti-Scl-70) antibodies are observed in the diffuse form (dcSSc) with a higher incidence of interstitial lung disease (ILD) (4, 5). However, autoantibodies are of limited use to predict the onset of specific complications (6–8); other biomarkers, such as uric acid and NT-ProBNP are instrumental to assess the risk of having PAH (9), KL-6 may predict the progression of SSc-ILD (10), while the combination of serum NT-ProBNP and troponins may be evocative of myocardial involvement (11, 12).

In recent years, proteomic analysis has been broadly used to identify biomarkers as it illustrated by the transcriptional profile changes associated with specific disease manifestations (13–19). Aptamers are short single-stranded oligonucleotides capable of folding into various structures, and have the ability to bind to proteins, peptides, and small molecules at concentrations ranging from femtomolar to micromolar, with high reproducibility and low variability rates (20) and holds promising in SSc to elucidate the molecular mechanisms of disease pathogenesis and internal organ involvement (21). We took advantage of proteomic aptamer analysis and different validation cohorts of SSc patients with and without ILD to identify biomarkers for SSc phenotypes.

## Methods

### Patients

Six patients who met the 2013 European League Against Rheumatism/American College of Rheumatology classification criteria for SSc (22) were enrolled from the Scleroderma Unit, Rheumatology and Clinical Immunology, IRCCS Humanitas Research Hospital in Rozzano, Italy. Clinical data were recorded, including organ involvement based on clinical features, laboratory results, and findings from diagnostic imaging (radiological imaging, echocardiography, lung function tests, and other relevant examinations). SSc-ILD was diagnosed based on high resolution computed tomography (HRCT) scans, following the guidelines set by the Fleischner Society (23). PAH was defined using right-heart catheterization in the presence of suspected/suggestive signs, symptoms, echocardiographic abnormalities, or pulmonary function abnormalities (24). Myocardial involvement was diagnosed based on compatible findings at cardiac magnetic resonance (25) in patients with suspected/suggestive signs or symptoms, electrocardiographic and 24-hour Holter ECG alterations, or elevated serum myocardial enzymes (11, 12). Serum autoantibodies were assessed using commercially available kits in routine laboratory analysis. Early SSc was defined as a disease duration less than 3 years from the onset of the first non-Raynaud manifestation (26). Patients were naïve to any immunosuppressive or vasoactive treatment. Seven healthy controls (HC) were enrolled (2 females, 5 males, median age 54 years, interquartile 46-67 years).

We also used two separate and independent cohorts of SSc patients and HC to validate our findings, as described below.

In all cases and controls, serum samples were collected using serum separator tubes, allowed to clot, aliquoted, and stored at -80° C.

This study was conducted in accordance with the Declaration of Helsinki, and the research protocol was approved by the local ethics committee (study number 831); all subjects provided their informed consent.

### Aptamer proteomic analysis

Serum aliquots of 150 μL were prepared from all collected samples and subjected to proteome profiling using a high throughput multiplexing aptamer-based SOMAscan^®^ assay, targeting 1310 serum proteins (Somalogic Inc., Boulder, CO), as previously described (27). The technique utilizes a panel of protein-specific Slow Off-rate Modified DNA aptamers (SOMAmers), which are constructed from chemically modified nucleotides capable of specifically recognizing and binding proteins with high specificity and affinity. The SOMAmer reagents are labeled with a 5’fluorophore and a biotin, immobilized on streptavidin coated beads, and incubated with serum samples. Complexes consisting of SOMAmer reagents and target proteins are formed on the beads. The SOMAmer reagents are then quantified through fluorescence using microarrays containing specific sequences. The relative intensity of fluorescence correlates to the amount of protein in the original sample.

### Validation with ELISA tests

Proteins that showed significantly different concentrations at the proteomic aptamer analysis (SomaSuite, *vide infra*) were selected for further validation and clinical correlations based on their pathogenic significance (as supported by previous literature) or if their relative fluorescence intensity was notably elevated. For validation, solid-phase enzyme immunoassays, ELISA tests, were employed to quantitatively determine the levels of the most relevant proteins in separate cohorts of SSc patients and HC. The following kits were used for ELISA testing: Human CD177, Eotaxin1, Leptin, Angiopoietin 2 (Ang2), Kininogen HMW, TPSB2, MMP12, IL-22BP (Raybiotech, USA), and Human Calpain 1, Aldolase A, BAFFR, Fractalkine, and Calgranulin B (S100 A9) (Mybiosuorce, Vancouver Canada), following the manufacturer’s instructions. All candidate proteins were first validated in an independent cohort of 30 patients with SSc and 10 HC (8 women, median age 71 years, interquartile range 62-74 years). Three proteins (Ang2, IL-22BP, and TPSB2) that exhibited statistical significance for multiple variables and displayed critical and innovative correlations with disease pathogenesis were further validated in an expanded cohort consisting of 89 patients with SSc and 43 HC (35 females, 8 males, median age 65 years, interquartile range 50-70 years).

### Statistical analysis

Data analysis of the SomaLogic results was conducted using SomaSuite software (Somalogic, Boulder, CO, USA). Statistical analysis of the ELISA data was performed using Stata16 software (StataCorp. 2019. Stata Statistical Software: Release 16. College Station, TX: StataCorp LLC). Non-parametric data were analyzed using the Wilcoxon rank-sum (Mann-Whitney) test for individual comparisons. To account for multiple comparisons, the Kruskal-Wallis correction was applied. A significance level of *p* < 0.05 was considered statistically significant. Variables that reached statistical significance in the univariate analysis were entered into a logistic regression analysis with both forward and backward stepwise selection procedure to identify independent risk factors for selected variables.

Pathway analysis was performed by Reactome peer-reviewed pathway database (https://reactome.org) through the over-representation analysis: a statistical test (hypergeometric distribution) that determines whether certain Reactome pathways are over-represented (enriched) in the submitted data. The probability score was corrected for false discovery rate using the Benjamani-Hochberg method.

## Results

### Patients

The characteristics of the three cohorts of patients, namely the SOMAscan, first ELISA validation, and extended ELISA validation groups, are illustrated in **Table 1**, with slight differences in terms of rarer organ involvements. Among the six patients studied with the SOMAscan proteomic aptamer analysis, all were female, with a median age of 64.5 years (interquartile range – IQR 41-73). One (17%) had dcSSc, 3 (50%) were ACA-positive, and 2 (33%) were anti-Scl-70 positive. Three patients (50%) had ILD and 5 (83%) had gastrointestinal involvement, while no patient had PAH.

**Table 1:** Demographic features of SSc patients analyzed by SomaLogic, the initial and extended validation cohorts.

	Aptamer cohort (n = 6)	First validation cohort (n = 30)	Extended validation cohort (n = 89)
Female, n (%)	6 (100)	26 (87)	82 (92)
Age, median (IQR)	64.5 (41-73)	63.5 (48-75)	64 (52-73.5)
dcSSc, n (%)	1 (17)	7 (23)	17 (19)
*Sine scleroderma*, n (%)	0 (0)	4 (13)	20 (22)
ACA, n (%)	3 (50)	14 (47)	47 (53)
Anti-Scl-70, n (%)	2 (33)	13 (43)	30 (34)
Anti-RNA polymerase III, n (%)	0 (0)	3 (10)	6 (7)
Early SSc, n (%)	3 (50)	14 (47)	38 (43)
ILD, n (%) dcSSc with ILD, n (%)	3 (50) 1 (17)	12 (40) 2 (7)	30 (34) 10 (11)
Cardiomyopathy, n (%)	1 (17)	6 (20)	17 (19)
PAH, n (%)	0 (0)	2 (7)	14 (16)
Gastrointestinal involvement, n (%)	5 (83)	14 (47)	43 (48)
Cancer, n (%)	1 (17)	5 (17)	14 (16)

ACA, anticentromere antibodies; dcSSc, diffuse cutaneous subset of SSc; ILD, interstitial lung disease; IQR, interquartile range; PAH, pulmonary arterial hypertension.

In the first validation cohort there were 30 patients, with a median age of 63.5 years (IQR 48-75). Seven (23%) had dcSSc, 14 (47%) were ACA-positive, and 13 (43%) were anti-Scl-70 positive. Additionally, 3 (10%) had anti-RNA polymerase III antibodies. Out of the 30 patients, 14 (47%) were in the early stage of SSc, 12 (40%) had ILD, 6 (20%) had cardiomyopathy, 14 (47%) had gastrointestinal involvement, and 2 (7%) had PAH.

The extended validation cohort consisted of 89 patients, with a median age of 64 years (IQR 52-73.5). Seventeen (19%) had dcSSc, 47 (53%) were ACA-positive, and 30 (34%) were anti-Scl-70 positive. Moreover, 6 (7%) had anti-RNA polymerase III antibodies. Among the 89 patients, 38 (43%) were in the early stage of SSc, 30 (34%) had ILD, 17 (19%) had cardiomyopathy, 43 (48%) had gastrointestinal involvement, and 14 (16%) had PAH.

### Proteomics of SSc and SSc-ILD using aptamers

**Figure 1:**
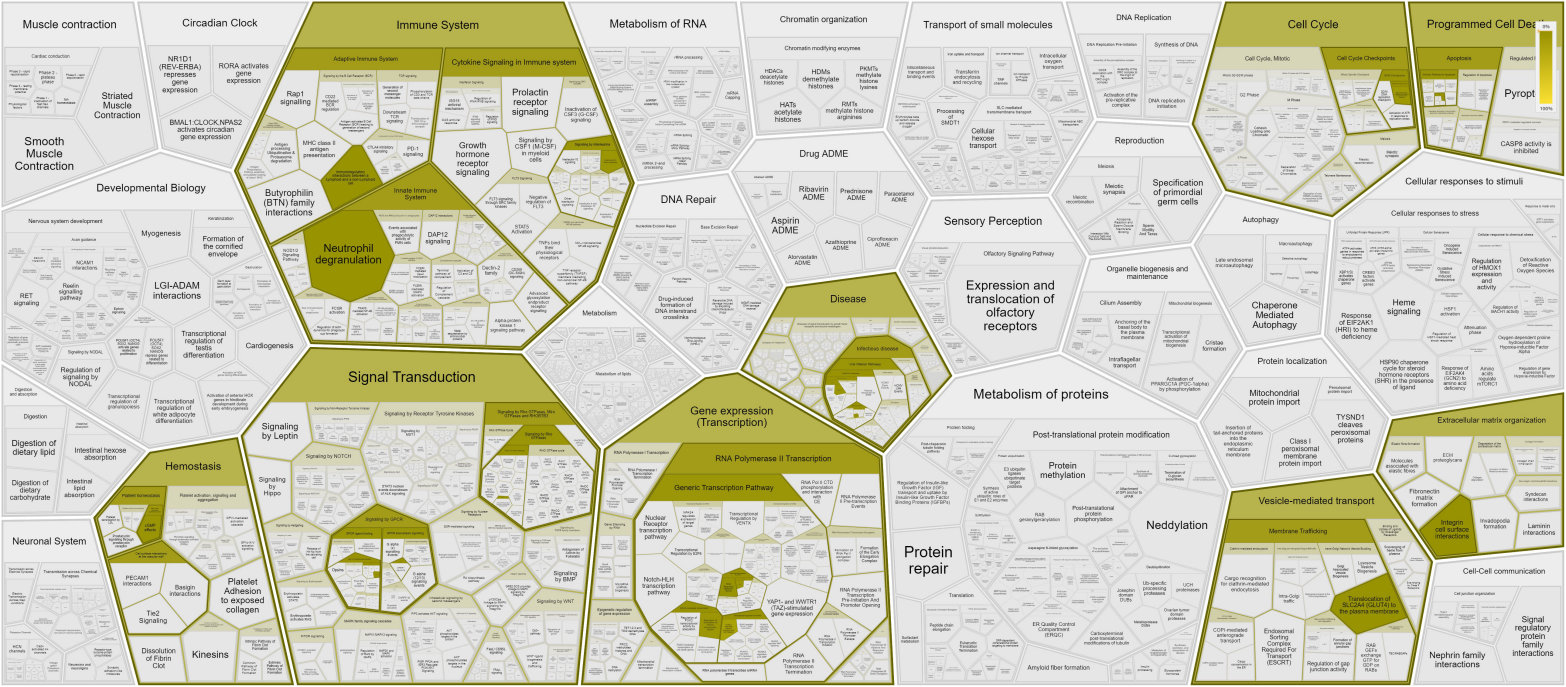
Pathways and interactions (https://reactome.org) of proteins showing significantly different (*p* < 0.05) serum levels in SSc patients compared with HC, as assessed using the aptamer (SomaLogic) proteomics platform.

Our first-tier proteomic analysis using SOMAscan technology revealed significant differences in serum levels of 33 proteins between patients with SSc and HC (**Table 2**). Patients with SSc exhibited altered expression of proteins involved in various biological processes, including extracellular matrix formation and cell-to-cell adhesion (elevated Calpain, EphA5, IDS, MATN2, MMP-12, TNR4, and reduced levels of desmoglein-1, SNP25), angiogenesis (increased levels of anti-angiogenic factors such as Ang2 and high molecular weight kininogen), lymphocyte recruitment, activation, and signaling (elevated levels of CXCL-1, LAG3 and decreased levels of SH21A), and overall inhibition of neutrophil function (decreased levels of G-CSF-R, CD177, calgranulin B; **Figure 1**). Nine proteins differentiated patients with SSc-ILD and without ILD or HC (**Table 3**). SSc-ILD patients showed elevated serum levels of proteins involved in intracellular signaling and cell cycle regulation (FCRL3, PDE11, Stratifin), as well as increased levels of MCP-3, a monocyte chemoattractant, and sICAM-5, the ligand for leukocyte adhesion protein LFA-1, compared to patients without ILD. Patients with SSc-ILD exhibited higher levels of IL-22BP, the decoy receptor for IL-22, and lower levels of BAFF.

**Table 2:** Significantly different (*p* < 0.05) protein serum levels in SSc patients compared with HC as assessed using the aptamer (SomaLogic) proteomics platform.

Increased in SSc	Reduced in SSc
Aldolase A Angiopoietin-2* C1QR1 Calpain COLEC12 Eotaxin EphA5 Fractalkine/CXCL-1 Granulins IDS Kininogen HMW LAG-3 Lamin-B1 LRP1b MATN2 MMP-12 STAT1 TNR4	Adrenomedullin ASGR1 C1s C5 Calgranulin B CD177 Desmoglein-1 Flt-3 ligand G-CFS-R IL-1Ra Leptin Lypd3 SH21A SNAP25 TPSB2

ASGR1, asialoglycoprotein receptor 1; C1QR1, complement component C1q receptor 1; C1s, complement fraction 1s; C5, complement fraction 5; COLEC12, collectin-12; EphA5, ephrin type-A receptor 5; G-CSF-R, receptor for granulocyte colony stimulating factor; HMW, high molecular weight; IDS, iduronate 2-sulfatase; IL-1Ra, interleukin-1 receptor antagonist; LAG-3, lymphocyte-activation gene 3; LRP1b, low-density lipoprotein receptor-related protein 1B; Lypd3, LY6/PLAUR domain containing 3; MATN2, matrilin 2; MMP-12, matrix metalloproteinase 12.

SH21A, SH2 domain-containing protein 1A; SNAP25, synaptosome associated protein 25; STAT1, signal transducer and activator of transcription 1; TNR4, TNF receptor superfamily member 4; TPSB2, tryptase beta-2.

* Significantly increased also at ELISA validation (p < 0.0001).

**Table 3:** Significantly different (*p* < 0.05) protein serum levels in SSc with ILD compared with SSc without ILD and HC as assessed using SomaLogic proteomics.

Increased in SSc-ILD	Reduced in SSc-ILD
FCRL3 IL-22BP** MCP-3 PDE11 PGP9.5 sICAM-5 Stratifin	BAFF DERM

BAFF, B cell activating factor; DERM, dermatopontin; FCRL3, Fc receptor-like protein 3; IL-22BP, interleukin-22 binding protein; MCP-3, human monocyte chemotactic protein-3; PDE11, phosphodiesterase 11; PGP9.5, protein gene product 9.5; sICAM-5, soluble intercellular adhesion molecule 1.

**Significantly increased also at ELISA validation comparing patients with SSc-ILD and HC (p < 0.0001).

### Validation of protein biomarkers and association with disease manifestations

In the second-tier analysis, we selected 13 proteins from the first analysis to be validated in the independent cohort (**Tables 4**–**7**).

**Table 4:** Serum concentration -median (IQR)- of validated proteins in HC, and SSc according to the presence or absence of SSc-ILD.

	HC	SSc	SSc-ILD	SSc without ILD
Aldolase A	71.9 (67.0-97.4)	91.9 (78.3-102)	92.6 (76.7-95.8)	88.4 (78.3-110)
Ang2	0.58 (0.34-0.82)*°	1.15 (0.60-2.41)*	1.45 (0.79-3.09)°	1.08 (0.58-2.12)
BAFFR	1.04 (0.38-1.28)	1.05 (0.64-2.38)	0.85 (0.58-1.54)	1.39 (0.80-2.62)
Calgranulin B	24.0 (11.6-45.4)	29.0 (16.6-65.0)	31.4 (21.7-60.1)	26.9 (16.6-65.0)
Calpain	12.7 (11.6-13.2)	13.4 (12.6-14.0)	13.0 (12.1-13.7)	13.6 (12.7-14.2)
CD177	12.3 (10.5-16.1)	10.9 (7.28-15.5)	10.4 (6.34-20.0)	10.9 (7.36-14.7)
Eotaxin	1.09 (0.74-1.65)	0.94 (0.62-1.35)	1.03 (0.56-1.36)	0.93 (0.62-1.35)
Fractalkine	2.37 (1.38-3.29)	2.82 (1.39-3.49)	2.37 (1.38-4.35)	2.87 (1.39-3.49)
IL-22BP	20.4 (13.9-37.6)°	26.2 (16.1-51.0)	36.3 (18.7-57.8)°	24.7 (13.7-50.8)
Kininogen	596 (506-765)	522 (352-707)	604 (299-807)	515 (433-689)
Leptin	2.11 (0.83-3.66)	1.92 (0.60-4.10)	2.09 (1.02-3.85)	1.92 (0.33-4.10)
MMP-12	0.20 (0.16-0.33)*°	0.10 (0.06-0.16)*	0.09 (0.05-0.13)°	0.12 (0.06-0.18)
TPSB2	42.6 (17.9-84.3)	59.6 (23.4-123)	67.5 (16.6-190)	56.9 (27.2-98.3)

HC, healthy controls; SSc-ILD, SSc-associated interstitial lung disease.

*p < 0.05 comparing SSc vs. HC.

°p < 0.05 comparing SSc-ILD vs. HC.

p > 0.05 comparing SSc without ILD vs HC.

**Table 5:** Serum concentration -median (IQR)- of validated proteins in SSc patients, according to the cutaneous subset.

	dcSSc	lcSSc	sine SSc	lcSSc + sine SSc
Aldolase A	85.5 (80.5-104)°	83.7 (74.0-95.5)°	123 (116-148)^°	92.5 (74.7-102.4)
Ang2	1.58 (0.80-2.72)	1.17 (0.66-2.39)	0.58 (0.37-1.65)	1.08 (0.58-2.12)
BAFFR	1.67 (0.80-3.20)	1.01 (0.60-1.84)	0.60 (0.34-1.62)	1.01 (0.56-1.84)
Calgranulin B	65.7 (62.2-81.8)*°	25.3 (16.5-33.1)°	49.0 (34.5-64.7)°	25.6 (16.6-40.8)*
Calpain	13.6 (12.7-14.7)	13.4 (12.4-14.0)	12.8 (12.5-14.4)	13.4 (12.4-14.0)
CD177	7.28 (4.97-14.7)	12.2 (8.79-19.9)	8.97 (3.68-10.9)	11.3 (8.17-19.9)
Eotaxin	1.35 (0.83-1.75)	0.95 (0.65-1.21)	0.55 (0.40-0.80)	0.94 (0.49-1.18)
Fractalkine	2.90 (1.07-3.32)	2.00 (1.35-4.01)	3.24 (2.89-4.72)	2.78 (1.39-4.08)
IL-22BP	24.9 (16.8-86.4)	27.8 (15.3-52.7)	23.6 (15.7-36.9)	26.6 (15.8-50.9)
Kininogen	505 (286-707)	591 (390-717)	384 (352-416)	564 (371-699)
Leptin	1.74 (0.79-3.00)	2.87 (0.77-4.92)	0.18 (0-1.21)^	2.09 (0.48-4.70)
MMP-12	0.07 (0.03-0.20)	0.13 (0.09-0.18)	0.05 (0.03-0.09)	0.11 (0.08-0.16)
TPSB2	23.5 (0-72.3)	71.7 (30.7-162)	64.6 (33.5-103)	71.7 (31.3-151)

dcSSc, diffuse cutaneous subset of SSc; lcSSc, limited cutaneous subset of SSc; sine SSc, SSc sine scleroderma.

*p < 0.05 comparing dcSSc vs. lcSSc and SSc sine scleroderma.

^p < 0.05 comparing SSc sine scleroderma vs. dcSSc and lcSSc.

°p < 0.05 comparing dcSSc vs. lcSSc vs. SSc sine scleroderma (three-group Kruskal-Wallis).

**Table 6:** Serum concentration -median (IQR)- of validated proteins in SSc patients, according to disease duration and organ involvement.

	Early SSc	Long SSc	Cardiomyopathy	PAH	GI
Aldolase A	97.6 (80.5-122)	83.8 (76.5-93.3)	87.8 (74.7-92.7)	132.8 (93.8-172)	91.9 (78.4-96.0)
Ang2	1.06 (0.56-1.65)°^	1.16 (0.65-3.46)^	1.28 (0.62-2.74)	1.38 (0.57-2.72)	1.25 (0.63-2.25)
BAFFR	1.08 (0.56-2.43)	1.05 (0.66-1.78)	1.25 (0.68-1.72)	0.94 (0.86-1.01)	1.36 (0.68-2.43)
Calgranulin B	36.9 (28.1-65.7)	25.3 (16.6-49.1)	33.1 (23.1-62.2)	57.3 (28.1-86.5)	30.7 (16.6-79.4)
Calpain	13.4 (12.7-14.0)	13.4 (12.1-14.1)	13.2 (12.7-13.9)	12.1 (11.8-12.4)	13.0 (12.4-13.9)
CD177	9.13 (6.24-11.3)*	12.3 (9.34-20.0)*	14.3 (6.15-19.9)	5.65 (0-11.3)	10.8 (6.15-12.4)
Eotaxin	0.91 (0.41-1.18)	1.01 (0.71-2.64)	0.80 (0.45-1.35)	0.85 (0.49-1.21)	1.16 (0.88-3.12)^#^
Fractalkine	2.90 (1.35-3.32)	2.66 (1.62-4.66)	3.27 (2.85-3.46)	3.90 (1.85-5.96)	1.62 (10.8-3.22)^#^
IL-22BP	26.2 (14.7-60.7)	26.4 (16.3-50.9)	28.2 (25.8-54.6)	28.3 (24.4-56.0)	27.5 (20.7-50.8)
Kininogen	501 (325-682)	579 (416-717)	638 (505-682)	310	591 (508-717)
Leptin	1.63 (0.48-2.93)	2.76 (0.69-4.81)	2.12 (1.02-2.65)	1.43 (0.77-2.09)	1.63 (0.60-5.45)
MMP-12	0.07 (0.03-0.10)*°^	0.15 (0.09-0.20)*^	0.14 (0.09-0.20)	0.03	0.13 (0.09-0.20)
TPSB2	48.0 (0-151)	66.2 (31.3-123)	65.7 (23.0-315)	71.7 (47.9-151)	67.9 (38.6-105)

GI, gastrointestinal involvement; HC, healthy controls; PAH, pulmonary arterial hypertension.

*p < 0.05 comparing early SSc vs. long standing disease.

°p < 0.05 comparing early SSc vs. HC.

^p < 0.05 comparing HC vs. early SSc vs. long standing disease (three-group Kruskal-Wallis).

^#^p < 0.05 comparing patients with and without gastrointestinal involvement (GI).

**Table 7:** Serum concentration -median (IQR)- of validated proteins in SSc patients, according to autoantibody profile.

	ACA	Scl-70	RNApol III
Aldolase A	83.8 (78.3-96.0)	95.5 (73.5-104)	92.7 (85.5-110)
Ang2	1.07 (0.63-1.59)	1.58 (0.58-3.03)	1.79 (0-7.89)
BAFFR	1.36 (0.68-1.84)	1.01 (0.64-2.43)	0.80 (0.34-1.08)
Calgranulin B	24.2 (15.2-33.1)*^§^^	62.2 (28.3-79.4)*^§#^	57.2 (20.3-65.0)*
Calpain	13.6 (12.8-14.0)	12.9 (12.4-13.6)	14.7 (10.7-15.8)
CD177	11.0 (8.79-14.0)	11.3 (6.24-15.8)	7.36 (7.28-22.1)
Eotaxin	1.03 (0.77-2.17)	0.88 (0.41-1.21)	0.62 (0.34-1.75)
Fractalkine	1.98 (1.31-3.46)	2.99 (1.85-3.98)	2.90 (2.78-7.50)
IL-22BP	25.9 (13.7-52.7)	31.8 (20.7-49.2)	22.4 (18.3-23.8)
Kininogen	604 (522-682)	390 (299-807)	416 (111-496)
Leptin	2.76 (0.48-4.92)	1.74 (1.28-2.93)	0.79 (0.33-11.1)
MMP-12	0.14 (0.10-0.20)*^	0.07 (0.04-0.11)	0.03 (0.03-0.09)
TPSB2	67.9 (29.2-92.8)	34.8 (14.1-153)	70.5 (0-305)

ACA, anticentromere antibodies; RNApol III, anti-RNA polymerase III antibodies.

*p < 0.05 comparing ACA vs. anti-Scl-70 vs. anti-RNA polymerase III (three-group Kruskal-Wallis).

^§^p < 0.05 comparing anti-Scl-70 vs. ACA.

^p < 0.05 comparing ACA vs. other autoantibodies.

^#^p < 0.05 comparing anti-Scl-70 vs. other autoantibodies.

We observed significantly higher levels of serum Ang2 in SSc, especially in SSc-ILD compared to HC [median (IQR): 1.15 ng/mL (0.60-2.41) in SSc, 1.45 ng/mL (0.79-3.09) in SSc-ILD, 0.58 ng/mL (0.34-0.82) in HC; *p* = 0.0001 for both comparisons]. IL-22BP was also present at higher concentrations in sera from patients with SSc-ILD [36.3 ng/mL (18.7-57.8)], with a progressive decrease in SSc without ILD [24.7 ng/mL (13.7-50.8)] and HC [20.4 ng/mL (13.9-37.6); *p* = 0.02 SSc-ILD vs. HC].

Concentrations of MMP-12 were significantly lower in SSc, particularly in SSc-ILD, compared to HC [0.10 ng/mL (0.06-0.16) in SSc, 0.09 ng/mL (0.05-0.13) in SSc-ILD, 0.20 ng/mL (0.16-0.33) in HC; *p* = 0.003 for SSc vs. HC and *p* = 0.01 for SSc-ILD vs. HC].

In terms of skin involvement, patients with SSc were stratified into three subsets (28), namely dcSSc, lcSSc, and SSc *sine scleroderma* (**Table 5**). Higher levels of calgranulin B were observed in dcSSc compared to other SSc forms [65.7 ng/mL (62.2-81.8) in dcSSc vs. 25.6 ng/mL (16.6-40.8) in the other subsets; *p* = 0.02]. In comparison to patients with skin involvement (i.e., dcSSc and lcSSc), patients with SSc *sine scleroderma* exhibited lower serum levels of leptin [0.18 ng/mL (0-1.21) in sine vs. 2.58 ng/mL (0.79-4.70) in the other subsets; *p* = 0.03], and higher levels of aldolase A [123 ng/mL (116-148) in sine vs. 84.7 ng/mL (74.7-96) in the other subsets; *p* = 0.003].

Results comparing disease duration (early SSc vs. long-standing disease) are illustrated in **Table 6**. Serum levels of MMP-12 were significantly lower in the early SSc group [0.07 ng/mL (0.03-0.10) in early SSc vs. 0.15 ng/mL (0.09-0.20) in long-standing disease; *p* = 0.003] and CD177 [9.13 ng/mL (6.24-11.3) in early SSc vs. 12.3 ng/mL (9.34-20.0) in long-standing disease; *p* = 0.03]. Multivariate analysis confirmed that MMP-12 is independently associated to early SSc [OR 5.7×10^-10^; 95% CI (7.2×10^-19^-0.45); *p* = 0.04].

Lower concentrations of fractalkine [1.62 ng/mL (10.8-3.22) vs. 2.95 ng/mL (2.77-4.61); *p* = 0.02] and higher concentrations of eotaxin [1.16 ng/mL (0.88-3.12) vs. 0.74 ng/mL (0.43-1.08); *p* = 0.03] were associated with gastrointestinal involvement, along with a non-significant increase in MMP-12 [0.13 ng/mL (0.09-0.20) vs. 0.09 ng/mL (0.03-0.14); *p* = 0.08] (**Table 6**). No significant differences were found when stratifying patients with SSc based on the presence of PAH or cardiomyopathy (**Table 6**).

We also compared patients based on autoantibody profiling, specifically ACA, anti-Scl-70, and anti-RNA polymerase III (**Table 7** **).** Patients with ACA had significantly higher levels of MMP-12 [0.14 ng/mL (0.10-0.20) for ACA vs. 0.07 ng/mL (0.04-0.11) for anti-Scl-70 vs. 0.03 ng/mL (0.03-0.09) for anti-RNA polymerase III; *p* = 0.01]. Calgranulin B showed the lowest values in ACA-positive subjects [24.2 ng/mL (15.2-33.1) for ACA vs. 59.7 ng/mL (28.2-75.7) for other antibodies; *p* = 0.01] and the highest levels with anti-Scl-70 [62.2 ng/mL (28.3-79.4) for anti-Scl-70 vs. 25.3 ng/mL (16.6-40.8) for other autoantibodies; *p* = 0.03].

## Discussion

The complex pathogenesis of SSc encompasses aberrant inflammation, dysregulated fibrosis, and microvascular disease (2). Comprehensive proteomic analysis is an ideal approach for identifying relevant molecules involved in various disease mechanisms, including potential prognostic factors, predictive molecules, and therapeutic targets. Proteomic analyses have already been performed and associated to clinical phenotype in SSc (29–32), as shown by the report that the higher expression of CXCL4 from peripheral blood and skin plasmacytoid dendritic cells in association with the incidence and progression of ILD and PAH in SSc (33). In addition, altered serum levels of collagen IV, endostatin, IGFBP-2, IGFBP-7, MMP-2, neuropilin-1, NT-proBNP, and RAGE have been described in SSc-PAH patients (34). Further, patients with a prominent signature based on CD40 ligand, CXCL4, and anti-PM/Scl-100 antibodies have shown a preferential positive response to treatment with the tyrosine kinase inhibitor imatinib (35).

Only a few studies have been conducted using the aptamer tools for proteomics in SSc (36–39). Within the studies investigating the preclinical phase of SSc (36, 39) one identified three proteins involved in the dysregulated angiogenesis and fibrosis being differentially expressed in patients with preclinical SSc at risk of evolving into overt disease, thus confirming the importance of microvascular disease in the earliest phases of SSc pathogenesis (36). Piera-Velazquez and Colleagues elegantly demonstrated that proteomic analysis of serum exosomes differs between patients with primary Raynaud’s phenomenon and patients with Raynaud’s phenomenon at risk of evolving into SSc (39). In the other two studies available (37, 38), the aptamer analysis was applied to describe longitudinal changes in patients with established SSc and organ damage. It was indeed demonstrated that serum levels of ST2 and spondin-1 predicted the changes in mRSS, also providing evidence of a peculiar cytokine signature (i.e., TNF, IFN-γ, TGF-β, and IL-13) (37). Additionally, chemerin was identified as a potential biomarker with pathogenic significance for increased pulmonary vascular resistances in patients with SSc-PAH (38). In another study, 82 proteins were found to be differentially expressed in sera from SSc-PAH patients compared to SSc patients without lung vascular involvement, including an IFN-γ signature and two other proteins of interest, Midkine (implicated in the pathogenesis of arterial hypertension, renal disease, and lung fibrosis) and Follistatin-like 3 (FSTL3, regulated by TGF-β) in association with SSc-PAH (40). A composite three-biomarker index (including Ca15-3, surfactant protein D, and ICAM-1) has been recently described to predict ILD in patients with SSc, and is associated to disease severity (41). Several reasons could explain the differences found in the proteomic profile between previous studies and our results. First, the types of samples, such as whole blood, serum, and exosomes, is expected to provide different analytical outcomes. Second, the correlation between the duration of the disease history and the timing of sample collection might have played a role, not only in the case of early vs. longstanding SSc but also in patients with isolated Raynaud’s phenomenon at risk of evolving towards established disease. Third, such results ultimately reflect the heterogeneity of SSc, indicating various internal organ involvements, as well as multiple nuances of disease severity and rates of disease progression.

Our multi-tier study including an aptamer-based analysis and a validation on independent cohorts supports the alterations in different biomarkers to reflect abnormal extracellular matrix formation, angiogenesis, vascular remodeling, and immune cell recruitment and function, which recapitulate the fundamental pathogenic aspects of SSc and some of the proposed biomarkers (Ang2, IL-22BP, and TPSB2) require a detailed discussion.

Angiopoietin-2 (Ang2) is a vascular growth factor secreted by endothelial cells that induces their own activation and promotes leukocyte chemotaxis. In the presence of a pro-inflammatory cytokine environment, this signaling pathway leads to vascular instability and endothelial inflammation (42). By stimulating the release of IL-6 and IL-8 from monocytes, Ang2 enhances the inflammatory-driven fibrogenic process, a hallmark of SSc (43). Our analysis revealed significantly increased serum levels of Ang2 in SSc patients compared to HC, which is consistent with previous literature findings (44). Furthermore, we observed significantly higher levels of serum Ang2 in early SSc and SSc-ILD patients compared to HC. Previous studies have shown that serum Ang2 levels decrease after treatment with intravenous cyclophosphamide in SSc-ILD patients, and this reduction correlates with concentrations of KL-6, an established biomarker of lung involvement (45). Overall, these results support the crucial role of aberrant angiogenesis across the pathogenic processes underlying SSc since the earliest phases, throughout the development of established disease, along with providing further support for the correlation between serum Ang2 concentrations and SSc-ILD.

IL-22 is an inflammatory cytokine produced by CD4+ T cells and innate T cells, including NKT, γδ T cells, and innate lymphoid cells (ILC) (46); IL-22BP is a soluble decoy receptor that acts as an IL-22 inhibitor (46). Ambivalent proinflammatory and modulating functions have been attributed to both IL-22 and IL-22BP in a tissue- and disease-dependent manner (47–49). A protective anti-inflammatory effect of IL-22 has been demonstrated in the case of pulmonary inflammation, with lower levels being detectable in the bronchoalveolar lavage fluid (BALF) of patients with acute respiratory distress syndrome and sarcoidosis (50). Moreover, IL-22 is essential to allow alveolar repair following Influenza pneumonia (51), while elevated IL-22BP expression increases the risk of severe pulmonary infections (52). On the other hand, a prominent IL-22-based inflammatory signature has been described in patients with SSc (53), with increased circulating Th22 cells (54) and serum IL-22 levels being associated with SSc-ILD (55). Expression of IL-22 in scleroderma skin is linked to both the inflammatory (56) and fibrotic responses that are responsible for disease progression (57). In our study, serum levels of IL-22BP were significantly increased in patients with SSc-ILD compared with HC. This points towards a role for reduced IL-22 function in the pathogenesis of SSc-ILD. A mouse model study reported that bleomycin-induced lung fibrosis leads to a decrease in IL-22, and administering exogenous IL-22 can inhibit the inflammatory and fibrotic process (58). We speculate that the protective role of IL-22 may be compromised in patients with SSc-ILD and modulating the IL-22/IL-22BP system could be a promising therapeutic target. Further studies are needed to clarify the role of cells producing IL-22 in the pathogenesis of scleroderma lung disease, with a particular focus on innate-like lymphocytes (59), which represent an intriguing crossroad between the environment, innate, and adaptive immunity.

Conflicting evidence has been reported regarding matrix metalloproteinase (MMP)-12, an enzyme with critical functions in extracellular matrix remodeling in animal models of lung fibrosis (60, 61). *In vitro* studies have shown that dermal fibroblasts from SSc patients overexpress MMP-12, thus affecting angiogenic homeostasis (62). Increased levels of MMP-12 in serum and tissue have been reported in SSc patients, and these levels are associated with longer disease duration and more severe skin and lung involvement (63). Furthermore, the rs2276109 polymorphism of the MMP-12 gene has been linked to SSc susceptibility in a large Italian cohort (64). Our results partially contrast with previous evidence. While we initially observed higher MMP-12 levels in SSc patients during SOMAscan analysis, we found significantly lower values during ELISA validation, especially in SSc-ILD, early SSc, and anti-Scl-70 or anti-RNA polymerase III positivity, suggesting a potential correlation between serum MMP-12 levels and milder forms of the disease. Such observations could suggest the presence of an ineffective extracellular matrix turnover, as reflected by lower levels of MMP-12, in those patients with a higher burden of fibro-inflammatory lesions. This is notably the case of individuals with SSc-ILD, as well as rapidly progressive cutaneous fibrosis associated with anti-RNA polymerase III antibodies. However, due to the discrepancy of previous evidence, further research is warranted also in this case to clarify these associations.

Dysfunction of the myeloid cell compartment has been implicated in both the inflammatory and fibrotic phases of SSc pathogenesis (65). To support this view, we found significant differences in serum concentrations of myeloid-derived proteins in SSc patients compared to HC, including calgranulin B, and CD177. Calgranulin B is a calcium-binding protein expressed in neutrophils, monocytes, and macrophages, and it is overexpressed in the lungs of patients with idiopathic pulmonary fibrosis and nonspecific interstitial pneumonia (66). Our analysis showed that SSc-ILD patients and dcSSc patients have higher circulating levels of calgranulin B. CD177 is a neutrophil membrane molecule involved in the regulation of diapedesis (67), and CD177+ neutrophils produce large amounts of IL-22 (68). As mentioned earlier, the ambivalent role of IL-22 may help explain the mild reduction in soluble CD177 that we observed in patients with early SSc. Further research is required to elucidate the role of the myeloid compartment in the pathogenesis of different subsets of SSc.

Two proteins associated with gastrointestinal involvement in patients with SSc are eotaxin and fractalkine. Eotaxin’s role in recruiting eosinophils and mast cells has been extensively studied in asthma (69), and its pro-fibrotic effects have been demonstrated in both animal models (70) and human conditions (71, 72). Recently, Piera-Velazquez and colleagues demonstrated that patients with early SSc have higher levels of eotaxin in circulating exosomes compared to subjects with primary Raynaud’s phenomenon (39). We are the first to report that increased serum levels of eotaxin and lower serum concentrations of fractalkine are significantly associated with esophageal involvement in patients with SSc.

Leptin warrants also a discussion as this is a metabolic hormone produced by adipose tissue cells and has potential, albeit conflicting, roles in autoimmune inflammation (73) and fibrosis (74). Its effects on the fibrotic process appear to be tissue- or organ-dependent (74). Contradictory data have been reported on serum leptin concentrations in patients with SSc (75–78), and it has been suggested that these variations may reflect heterogeneity in disease duration, activity, and different phenotypes and endotypes. We are the first to report low levels of leptin in patients with SSc *sine scleroderma* compared to subjects with cutaneous involvement (both lcSSc and dcSSc).

To our knowledge, this is the first study to investigate serum proteins using proteomic aptamer analysis in a deeply phenotyped and endotyped cohort of SSc patients to assess the potential pathogenetic roles, spanning from extracellular matrix formation, angiogenesis, and immune cell homing and function. The strength of our study is that patient sera were obtained at the time of diagnosis, prior to any immunosuppressive or vasoactive treatment initiation, therefore our findings are expected to accurately represent the serum proteome of treatment-naïve individuals with SSc. Functionality assays, including gene expression and epigenetic studies, could serve as powerful tools to test and enhance the pathogenic validity of our observations. For instance, by studying the modulation of angiogenic pathways (mainly represented by Ang2 in our dataset), extracellular matrix remodeling (such as MMP-12), or myeloid cell function, we could gain a deeper understanding of whether these processes play a “disease-modifying” role at various stages of the disease or in different organs. Silencing IL-22 in mice-models of SSc lung disease may prove helpful to understand if IL-22 has a different role in the lung compared to the skin. Functionality analysis could reveal critical and potentially practice-changing information while the checkpoint driving dysregulated fibrosis could be intercepted, or it might be revealed that targeting certain pathways (e.g., aberrant angiogenesis, lymphocyte activation) is crucial but only in certain disease phases. Among the limitations of our study, the small sample size used for the aptamer analysis and the validation performed on a larger cohort should be noted, as well as the arbitrary choice of the candidate proteins, based on their presumed pathogenic significance and available data from previous literature. Furthermore, while the composition of the validation cohorts adequately reflects the distribution of different disease subsets and organ manifestations, this is not the case for the SOMAscan cohort due to the low prevalence of dcSSc, absence of subjects with SSc *sine scleroderma*, anti-RNA polymerase III antibodies, and PAH.

## Conclusions

Serum and tissue proteomics offer valuable tools for characterizing various aspects of the disease, aligning with the principles of precision medicine, while prospective validation of these biomarkers is warranted. The potential biomarkers that distinguish patients with SSc from HC identified in this work play functional roles in extracellular matrix metabolism, angiogenesis, and immune cell function, which are critical checkpoints in the pathogenesis of the disease. Biomarkers related to altered angiogenesis can differentiate patients with early SSc from HC, while other molecules exhibit differential expression in patients with SSc depending on factors such as disease subset, autoantibody profile, extent of skin fibrosis, and internal organ involvement, including ILD.

## Data availability statement

The raw data supporting the conclusions of this article will be made available by the authors, without undue reservation.

## Ethics statement

The studies involving humans were approved by IRCCS Humanitas Research Hospital ethics committee. The studies were conducted in accordance with the local legislation and institutional requirements. The participants provided their written informed consent to participate in this study.

## Author contributions

Conceptualization and coordination: MS and CS. Laboratory investigations: NI, AC. Data collection and statistical analysis: FM, AT, MS, AC, and NI. Literature review: FM, AT, and GC. Manuscript preparation—original draft: FM and AT; supervision: MS and CS. All authors contributed to the article and approved the submitted version.

## References

[1] Gabrielli A Avvedimento EV Krieg T. Scleroderma. N Engl J Med (2009) 360(19):1989–2003. doi: 10.1056/NEJMra0806188 19420368

[2] Denton CP Khanna D. Systemic sclerosis. Lancet (2017) 390(10103):1685–99. doi: 10.1016/S0140-6736(17)30933-9 28413064

[3] Volkmann ER Andréasson K Smith V. Systemic sclerosis. Lancet (2023) 401(10373):304–18. doi: 10.1016/S0140-6736(22)01692-0 PMC9892343 36442487

[4] Kowal-Bielecka O Fransen J Avouac J Becker M Kulak A Allanore Y. Update of EULAR recommendations for the treatment of systemic sclerosis. Ann Rheum Dis (2017) 76(8):1327–39. doi: 10.1136/annrheumdis-2016-209909 27941129

[5] Roofeh D Jaafar S Vummidi D Khanna D. Management of systemic sclerosis-associated interstitial lung disease. Curr Opin Rheumatol (2019) 31(3):241–9. doi: 10.1097/BOR.0000000000000592 PMC6647025 30870216

[6] Skaug B Assassi S. Biomarkers in systemic sclerosis. Curr Opin Rheumatol (2019) 31(6):595–602. doi: 10.1097/BOR.0000000000000656 31436584 PMC7185900

[7] Cavazzana I Vojinovic T Airo’ P Fredi M Ceribelli A Pedretti E. Systemic sclerosis-specific antibodies: novel and classical biomarkers. Clin Rev Allergy Immunol (2023) 64(3):412–30. doi: 10.1007/s12016-022-08946-w PMC10167150 35716254

[8] Domsic RT. Scleroderma: the role of serum autoantibodies in defining specific clinical phenotypes and organ system involvement. Curr Opin Rheumatol (2014) 26(6):646–52. doi: 10.1097/BOR.0000000000000113 PMC4299717 25203118

[9] Coghlan JG Denton CP Grünig E Bonderman D Distler O Khanna D. Evidence-based detection of pulmonary arterial hypertension in systemic sclerosis: the DETECT study. Ann Rheum Dis (2014) 73(7):1340–9. doi: 10.1136/annrheumdis-2013-203301 PMC4078756 23687283

[10] Watanabe S Kase K Saeki K Ohkura N Murata A Waseda Y. Kinetic changes in serum KL-6 levels predict disease progression in patients with systemic sclerosis-associated interstitial lung disease. Respir Med (2022) 191:106689. doi: 10.1016/j.rmed.2021.106689 34844174

[11] Bosello S De Luca G Berardi G Canestrari G de Waure C Gabrielli FA. Cardiac troponin T and NT-proBNP as diagnostic and prognostic biomarkers of primary cardiac involvement and disease severity in systemic sclerosis: A prospective study. Eur J Intern Med (2019) 60:46–53. doi: 10.1016/j.ejim.2018.10.013 30366614

[12] Bosello S De Luca G Ferraccioli G. Troponin in stable ischemic heart disease and diabetes. N Engl J Med (2015) 373(20):1977–8. doi: 10.1056/NEJMc1511645 26559581

[13] Lourido L Blanco FJ Ruiz-Romero C. Defining the proteomic landscape of rheumatoid arthritis: progress and prospective clinical applications. Expert Rev Proteomics (2017) 14(5):431–44. doi: 10.1080/14789450.2017.1321481 28425787

[14] Butt S Jeppesen JL Iversen LV Fenger M Eugen-Olsen J Andersson C. Association of soluble urokinase plasminogen activator receptor levels with fibrotic and vascular manifestations in systemic sclerosis. PloS One (2021) 16(2):e0247256. doi: 10.1371/journal.pone.0247256 33617568 PMC7899346

[15] Chularojanamontri L Charoenpipatsin N Silpa-Archa N Wongpraparut C Thongboonkerd V. Proteomics in psoriasis. Int J Mol Sci (2019) 20(5):1141. doi: 10.3390/ijms20051141 30845706 PMC6429319

[16] Ling HZ Xu SZ Leng RX Wu J Pan HF Fan YG. Discovery of new serum biomarker panels for systemic lupus erythematosus diagnosis. Rheumatol (Oxford) (2020) 59(6):1416–25. doi: 10.1093/rheumatology/kez634 31899518

[17] Cecchettini A Finamore F Puxeddu I Ferro F Baldini C. Salivary extracellular vesicles versus whole saliva: new perspectives for the identification of proteomic biomarkers in Sjögren’s syndrome. Clin Exp Rheumatol (2019) 37 Suppl 118(3):240–8. 31464680

[18] Landi C Bargagli E Bianchi L Gagliardi A Carleo A Bennett D. Towards a functional proteomics approach to the comprehension of idiopathic pulmonary fibrosis, sarcoidosis, systemic sclerosis and pulmonary Langerhans cell histiocytosis. J Proteomics (2013) 83:60–75. doi: 10.1016/j.jprot.2013.03.006 23528693

[19] Szél E Bozó R Hunyadi-Gulyás É Manczinger M Szabó K Kemény L. Comprehensive proteomic analysis reveals intermediate stage of non-lesional psoriatic skin and points out the importance of proteins outside this trend. Sci Rep (2019) 9(1):11382. doi: 10.1038/s41598-019-47774-5 31388062 PMC6684579

[20] Gramolini A Lau E Liu PP. Identifying low-abundance biomarkers: aptamer-based proteomics potentially enables more sensitive detection in cardiovascular diseases. Circulation (2016) 134(4):286–9. doi: 10.1161/CIRCULATIONAHA.116.022940 27444931

[21] Wermuth PJ Piera-Velazquez S Jimenez SA. Identification of novel systemic sclerosis biomarkers employing aptamer proteomic analysis. Rheumatol (Oxford) (2018) 57(10):1698–706. doi: 10.1093/rheumatology/kex404 29140474

[22] van den Hoogen F Khanna D Fransen J Johnson SR Baron M Tyndall A. 2013 classification criteria for systemic sclerosis: an American college of rheumatology/European league against rheumatism collaborative initiative. Ann Rheum Dis (2013) 72(11):1747–55. doi: 10.1136/annrheumdis-2013-204424 24092682

[23] Hansell DM Bankier AA MacMahon H McLoud TC Müller NL Remy J. Fleischner Society: glossary of terms for thoracic imaging. Radiology (2008) 246(3):697–722. doi: 10.1148/radiol.2462070712 18195376

[24] Hassoun PM. Pulmonary arterial hypertension. N Engl J Med (2021) 385(25):2361–76. doi: 10.1056/NEJMra2000348 34910865

[25] Mavrogeni S Pepe A Gargani L Bruni C Quaia E Kitas GD. Cardiac inflammation and fibrosis patterns in systemic sclerosis, evaluated by magnetic resonance imaging: An update. Semin Arthritis Rheumatol (2022) 58:152126. doi: 10.1016/j.semarthrit.2022.152126 36434895

[26] Allanore Y Simms R Distler O Trojanowska M Pope J Denton CP. Systemic sclerosis. Nat Rev Dis Primers (2015) 1:15002. doi: 10.1038/nrdp.2015.2 27189141

[27] Lollo B Steele F Gold L. Beyond antibodies: new affinity reagents to unlock the proteome. Proteomics (2014) 14(6):638–44. doi: 10.1002/pmic.201300187 24395722

[28] LeRoy EC Black C Fleischmajer R Jablonska S Krieg T Medsger TA. Scleroderma (systemic sclerosis): classification, subsets and pathogenesis. J Rheumatol (1988) 15(2):202–5. 3361530

[29] Dumit VI Küttner V Käppler J Piera-Velazquez S Jimenez SA Bruckner-Tuderman L. Altered MCM protein levels and autophagic flux in aged and systemic sclerosis dermal fibroblasts. J Invest Dermatol (2014) 134(9):2321–30. doi: 10.1038/jid.2014.69 PMC4121389 24496236

[30] Aden N Shiwen X Aden D Black C Nuttall A Denton CP. Proteomic analysis of scleroderma lesional skin reveals activated wound healing phenotype of epidermal cell layer. Rheumatol (Oxford) (2008) 47(12):1754–60. doi: 10.1093/rheumatology/ken370 18829709

[31] Zian Z Bakkach J Barakat A Ghailani Nourouti N Bennani Mechita M. Salivary biomarkers in systemic sclerosis disease. BioMed Res Int (2018) 2018:3921247. doi: 10.1155/2018/3921247 29721505 PMC5867662

[32] Landi C Bargagli E Carleo A Refini RM Bennett D Bianchi L. Bronchoalveolar lavage proteomic analysis in pulmonary fibrosis associated with systemic sclerosis: S100A6 and 14-3-3ϵ as potential biomarkers. Rheumatol (Oxford) (2019) 58(1):165–78. doi: 10.1093/rheumatology/key223 30239835

[33] van Bon L Affandi AJ Broen J Christmann RB Marijnissen RJ Stawski L. Proteome-wide analysis and CXCL4 as a biomarker in systemic sclerosis. N Engl J Med (2014) 370(5):433–43. doi: 10.1056/NEJMoa1114576 PMC4040466 24350901

[34] Bauer Y de Bernard S Hickey P Ballard K Cruz J Cornelisse P. Identifying early pulmonary arterial hypertension biomarkers in systemic sclerosis: machine learning on proteomics from the DETECT cohort. Eur Respir J (2021) 57(6):2002591. doi: 10.1183/13993003.02591-2020 33334933 PMC8276065

[35] Haddon DJ Wand HE Jarrell JA Spiera RF Utz PJ Gordon JK. Proteomic analysis of sera from individuals with diffuse cutaneous systemic sclerosis reveals a multianalyte signature associated with clinical improvement during imatinib mesylate treatment. J Rheumatol (2017) 44(5):631–8. doi: 10.3899/jrheum.160833 PMC5860882 28298564

[36] Bellocchi C Assassi S Lyons M Marchini M Mohan C Santaniello A. Proteomic aptamer analysis reveals serum markers that characterize preclinical systemic sclerosis (SSc) patients at risk for progression toward definite SSc. Arthritis Res Ther (2023) 25(1):15. doi: 10.1186/s13075-023-02989-w 36707842 PMC9881382

[37] Rice LM Mantero JC Stifano G Ziemek J Simms RW Gordon J. A proteome-derived longitudinal pharmacodynamic biomarker for diffuse systemic sclerosis skin. J Invest Dermatol (2017) 137(1):62–70. doi: 10.1016/j.jid.2016.08.027 27640094

[38] Sanges S Rice L Tu L Valenzi E Cracowski JL Montani D. Biomarkers of haemodynamic severity of systemic sclerosis-associated pulmonary arterial hypertension by serum proteome analysis. Ann Rheum Dis (2023) 82(3):365–73. doi: 10.1136/ard-2022-223237 PMC9918672 36600187

[39] Piera-Velazquez S Dillon ST Gu X Libermann TA Jimenez SA. Aptamer proteomics of serum exosomes from patients with Primary Raynaud’s and patients with Raynaud’s at risk of evolving into Systemic Sclerosis. PloS One (2022) 17(12):e0279461. doi: 10.1371/journal.pone.0279461 36548367 PMC9779033

[40] Rice LM Mantero JC Stratton EA Warburton R Roberts K Hill N. Serum biomarker for diagnostic evaluation of pulmonary arterial hypertension in systemic sclerosis. Arthritis Res Ther (2018) 20(1):185. doi: 10.1186/s13075-018-1679-8 30115106 PMC6097341

[41] Jee AS Stewart I Youssef P Adelstein S Lai D Hua S. A composite serum biomarker index for the diagnosis of systemic sclerosis-associated interstitial lung disease: A multicenter, observational cohort study. Arthritis Rheumatol (2023) 75(8):1424–33. doi: 10.1002/art.42491 36908055

[42] Wu Q Xu WD Huang AF. Role of angiopoietin-2 in inflammatory autoimmune diseases: A comprehensive review. Int Immunopharmacol (2020) 80:106223. doi: 10.1016/j.intimp.2020.106223 31991374

[43] Carvalheiro T Lopes AP van der Kroef M Malvar-Fernandez B Rafael-Vidal C Hinrichs AC. Angiopoietin-2 promotes inflammatory activation in monocytes of systemic sclerosis patients. Int J Mol Sci (2020) 21(24):9544. doi: 10.3390/ijms21249544 33333969 PMC7765391

[44] Michalska-Jakubus M Kowal-Bielecka O Chodorowska G Bielecki M Krasowska D. Angiopoietins-1 and -2 are differentially expressed in the sera of patients with systemic sclerosis: high angiopoietin-2 levels are associated with greater severity and higher activity of the disease. Rheumatol (Oxford) (2011) 50(4):746–55. doi: 10.1093/rheumatology/keq392 21149250

[45] Takahashi T Asano Y Akamata K Aozasa N Taniguchi T Noda S. Dynamics of serum angiopoietin-2 levels correlate with efficacy of intravenous pulse cyclophosphamide therapy for interstitial lung disease associated with systemic sclerosis. Mod Rheumatol (2013) 23(5):884–90. doi: 10.3109/s10165-012-0755-1 22972016

[46] Dudakov JA Hanash AM van den Brink MRM. Interleukin-22: immunobiology and pathology. Annu Rev Immunol (2015) 33:747–85. doi: 10.1146/annurev-immunol-032414-112123 PMC4407497 25706098

[47] Zenewicz LA. IL-22 binding protein (IL-22BP) in the regulation of IL-22 biology. Front Immunol (2021) 12:766586. doi: 10.3389/fimmu.2021.766586 34868019 PMC8634938

[48] Voglis S Moos S Kloos L Wanke F Zayoud M Pelczar P. Regulation of IL-22BP in psoriasis. Sci Rep (2018) 8(1):5085. doi: 10.1038/s41598-018-23510-3 29572462 PMC5865214

[49] Pelczar P Witkowski M Perez LG Kempski J Hammel AG Brockmann L. A pathogenic role for T cell-derived IL-22BP in inflammatory bowel disease. Science (2016) 354(6310):358–62. doi: 10.1126/science.aah5903 27846573

[50] Whittington HA Armstrong L Uppington KM Millar AB. Interleukin-22: a potential immunomodulatory molecule in the lung. Am J Respir Cell Mol Biol (2004) 31(2):220–6. doi: 10.1165/rcmb.2003-0285OC 15039135

[51] Pociask DA Scheller EV Mandalapu S McHugh KJ Enelow RI Fattman CL. IL-22 is essential for lung epithelial repair following influenza infection. Am J Pathol (2013) 182(4):1286–96. doi: 10.1016/j.ajpath.2012.12.007 PMC3620404 23490254

[52] Hebert KD Mclaughlin N Galeas-Pena M Zhang Z Eddens T Govero A. Targeting the IL-22/IL-22BP axis enhances tight junctions and reduces inflammation during influenza infection. Mucosal Immunol (2020) 13(1):64–74. doi: 10.1038/s41385-019-0206-9 31597930 PMC6917921

[53] Mathian A Parizot C Dorgham K Trad S Arnaud L Larsen M. Activated and resting regulatory T cell exhaustion concurs with high levels of interleukin-22 expression in systemic sclerosis lesions. Ann Rheum Dis (2012) 71(7):1227–34. doi: 10.1136/annrheumdis-2011-200709 22696687

[54] Truchetet ME Brembilla NC Montanari E Allanore Y Chizzolini C. Increased frequency of circulating Th22 in addition to Th17 and Th2 lymphocytes in systemic sclerosis: association with interstitial lung disease. Arthritis Res Ther (2011) 13(5):R166. doi: 10.1186/ar3486 21996293 PMC3308100

[55] Kardum Ž Milas-Ahić J Šahinović I Masle AM Uršić D Kos M. Serum levels of interleukin 17 and 22 in patients with systemic sclerosis: a single-center cross-sectional study. Rheumatol Int (2023) 43(2):345–54. doi: 10.1007/s00296-022-05250-w 36416900

[56] Brembilla NC Dufour AM Alvarez M Hugues S Montanari E Truchetet ME. IL-22 capacitates dermal fibroblast responses to TNF in scleroderma. Ann Rheum Dis (2016) 75(9):1697–705. doi: 10.1136/annrheumdis-2015-207477 26452537

[57] Sawamura S Jinnin M Inoue K Yamane K Honda N Kajihara I. Regulatory mechanisms of collagen expression by interleukin-22 signaling in scleroderma fibroblasts. J Dermatol Sci (2018) 90(1):52–9. doi: 10.1016/j.jdermsci.2017.12.017 29336866

[58] Qu Z Dou W Zhang K Duan L Zhou D Yin S. IL-22 inhibits bleomycin-induced pulmonary fibrosis in association with inhibition of IL-17A in mice. Arthritis Res Ther (2022) 24:280. doi: 10.1186/s13075-022-02977-6 36564791 PMC9789559

[59] Van Kaer L Postoak JL Song W Wu L. Innate and innate-like effector lymphocytes in health and disease. J Immunol (2022) 209(2):199–207. doi: 10.4049/jimmunol.2200074 35821102 PMC9285656

[60] Matute-Bello G Wurfel MM Lee JS Park DR Frevert CW Madtes DK. Essential role of MMP-12 in Fas-induced lung fibrosis. Am J Respir Cell Mol Biol (2007) 37(2):210–21. doi: 10.1165/rcmb.2006-0471OC PMC1976544 17446527

[61] Manoury B Nenan S Guenon I Boichot E Planquois JM Bertrand CP. Macrophage metalloelastase (MMP-12) deficiency does not alter bleomycin-induced pulmonary fibrosis in mice. J Inflammation (Lond) (2006) 3:2. doi: 10.1186/1476-9255-3-2 PMC1397817 16504062

[62] Serratì S Cinelli M Margheri F Guiducci S Del Rosso A Pucci M. Systemic sclerosis fibroblasts inhibit in *vitro* angiogenesis by MMP-12-dependent cleavage of the endothelial cell urokinase receptor. J Pathol (2006) 210(2):240–8. doi: 10.1002/path.2048 16917801

[63] Manetti M Guiducci S ROmano E Bellando-Randone S Conforti ML Ibba-Manneschi L. Increased serum levels and tissue expression of matrix metalloproteinase-12 in patients with systemic sclerosis: correlation with severity of skin and pulmonary fibrosis and vascular damage. Ann Rheum Dis (2012) 71(6):1064–72. doi: 10.1136/annrheumdis-2011-200837 22258486

[64] Manetti M Ibba-Manneschi L Fatini C Guiducci S Cuomo G Bonino C. Association of a functional polymorphism in the matrix metalloproteinase-12 promoter region with systemic sclerosis in an Italian population. J Rheumatol (2010) 37(9):1852–7. doi: 10.3899/jrheum.100237 20595276

[65] Kania G Rudnik M Distler O. Involvement of the myeloid cell compartment in fibrogenesis and systemic sclerosis. Nat Rev Rheumatol (2019) 15(5):288–302. doi: 10.1038/s41584-019-0212-z 30953037

[66] Bennett D Salvini M Fui A Cillis G Cameli P Mazzei MA. Calgranulin B and KL-6 in bronchoalveolar lavage of patients with IPF and NSIP. Inflammation (2019) 42(2):463–70. doi: 10.1007/s10753-018-00955-2 30680696

[67] Bai M Grieshaber-Bouyer R Wang J Schmider AB Wilson ZS Zeng L. CD177 modulates human neutrophil migration through activation-mediated integrin and chemoreceptor regulation. Blood (2017) 130(19):2092–100. doi: 10.1182/blood-2017-03-768507 PMC5680608 28807980

[68] Zhou G Yu L Fang L Yang W Yu T Miao Y. CD177+ neutrophils as functionally activated neutrophils negatively regulate IBD. Gut (2018) 67(6):1052–63. doi: 10.1136/gutjnl-2016-313535 28468761

[69] Pease JE Williams TJ. Eotaxin and asthma. Curr Opin Pharmacol (2001) 1(3):248–53. doi: 10.1016/S1471-4892(01)00044-3 11712747

[70] Zweifel M Matozan K Dahinden C Schaffner T Mohacsi P. Eotaxin/CCL11 levels correlate with myocardial fibrosis and mast cell density in native and transplanted rat hearts. Transplant Proc (2010) 42(7):2763–6. doi: 10.1016/j.transproceed.2010.05.152 20832583

[71] Mari A Kadah A Mahamid M Sbeit W Khoury T. IgG4 related autoimmune pancreatitis: an overview and the emerging role of serum eotaxin as a potential treatment target. Isr Med Assoc J (2019) 21(9):620–3. 31542909

[72] Cheng E Zhang X Wilson KS Wang DH Park JY Huo X. JAK-STAT6 pathway inhibitors block eotaxin-3 secretion by epithelial cells and fibroblasts from esophageal eosinophilia patients: promising agents to improve inflammation and prevent fibrosis in eoe. PloS One (2016) 11(6):e0157376. doi: 10.1371/journal.pone.0157376 27310888 PMC4911010

[73] La Cava A. Leptin in inflammation and autoimmunity. Cytokine (2017) 98:51–8. doi: 10.1016/j.cyto.2016.10.011 PMC5453851 27916613

[74] Liu Y Li Y Liang J Sun Z Wu Q Liu Y. Leptin: an entry point for the treatment of peripheral tissue fibrosis and related diseases. Int Immunopharmacol (2022) 106:108608. doi: 10.1016/j.intimp.2022.108608 35180626

[75] Michalska-Jakubus M Sawicka K Potembska E Kowal M Krasowska D. Clinical associations of serum leptin and leptin/adiponectin ratio in systemic sclerosis. Postepy Dermatol Alergol (2019) 36(3):325–38. doi: 10.5114/ada.2018.75809 PMC6640022 31333350

[76] Lee YH Song GG. Meta-analysis of circulating adiponectin, leptin, and resistin levels in systemic sclerosis. Z Rheumatol (2017) 76(9):789–97. doi: 10.1007/s00393-016-0172-5 27515182

[77] Budulgan M Dilek B Dağ ŞB Batmaz I Yıldız İ Sarıyıldız MA. Relationship between serum leptin level and disease activity in patients with systemic sclerosis. Clin Rheumatol (2014) 33(3):335–9. doi: 10.1007/s10067-013-2459-0 24370646

[78] Pehlivan Y Onat AM Ceylan N Turkbeyler IH Buyukhatipoglu H Comez G. Serum leptin, resistin and TNF-α levels in patients with systemic sclerosis: the role of adipokines in scleroderma. Int J Rheum Dis (2012) 15(4):374–9. doi: 10.1111/j.1756-185X.2012.01755.x 22898217

